# Surface Assessment of a Novel Acid-Etching Solution on CAD/CAM Dental Ceramics

**DOI:** 10.3390/biomimetics10080508

**Published:** 2025-08-04

**Authors:** Fabio Andretti, Carlos A. Jurado, Mark Antal, Alfredo I. Hernandez, Silvia Rojas-Rueda, Franklin Garcia-Godoy, Brian R. Morrow, Hamid Nurrohman

**Affiliations:** 1 Division of Oper Dent, Department of General Dentistry, College of Dentistry, The University of Tennessee Health Science Center, Memphis, TN 38104, USA; fandrett@uthsc.edu; 2 School of Dental Medicine, Ponce Health Sciences University, Ponce 00732, Puerto Rico; 3 Department of Operative and Esthetic Dentistry, Faculty of Dentistry, University of Szeged, 6720 Szeged, Hungary; 4 Prosthodontics Department, Arizona School of Dentistry and Oral Health, A.T. Still University, Mesa, AZ 85206, USA; alfredohernandez@atsu.edu; 5 Division of Dental Biomaterials, Department of Clinical and Community Sciences, School of Dentistry, The University of Alabama at Birmingham, Birmingham, AL 35233, USA; 6 Department of Bioscience Research, College of Dentistry, The University of Tennessee Health Science Center, Memphis, TN 38163, USA; 7 Department of Restorative Dentistry and Prosthodontics, The University of Texas School of Dentistry, Houston, TX 77054, USA

**Keywords:** dental ceramic, multi-acid etching, surface properties

## Abstract

Background: This study investigated a new multi-acid-etching formulation for zirconia ceramics, containing hydrochloric, hydrofluoric, nitric, orthophosphoric, and sulfuric acids. The solution was tested on polycrystalline (5Y-TZP zirconia), lithium disilicate, hybrid ceramic, and feldspathic porcelain to assess compatibility, etching selectivity, and surface conditioning. Methods: Two-hundred-and-forty CAD/CAM specimens were etched for 20 s, 60 s, 30 min, or 1 h, and their surface roughness and etching patterns ware evaluated using 3D optical profilometry and scanning electron microscopy (SEM). Results: A positive correlation was observed between etching time and surface roughness (Ra values). The most pronounced changes were observed in lithium disilicate and feldspathic porcelain, with Ra values increasing from 0.733 ± 0.082 µm (Group 5) to 1.295 ± 0.123 µm (Group 8), and from 0.902 ± 0.102 µm (Group 13) to 1.480 ± 0.096 µm (Group 16), respectively. Zirconia increased from 0.181 ± 0.043 µm (Group 1) to 0.371 ± 0.074 µm (Group 4), and the hybrid ceramic from 0.053 ± 0.008 µm (Group 9) to 0.099 ± 0.016 µm (Group 12). Two-way ANOVA revealed significant effects of material and etching time, as well as a significant interaction between the two factors (*p* < 0.001). SEM observation revealed non-selective etching pattern for the lithium disilicate groups, indicating a risk of over-etching. Conclusions: The tested etching solution increased surface roughness, especially for the lithium disilicate and feldspathic porcelain specimens. In zirconia, one-hour etching improved surface characteristics with minimal observable damage. However, additional studies are necessary to validate the mechanical stability and bond effectives of this approach.

## 1. Introduction

Dental ceramics have undergone substantial advancements, providing clinicians with a diverse range of materials that vary significantly in composition, microstructure, and clinical indications. Lithium disilicate ceramics, composed primarily of needle-like Li_2_Si_2_O_5_ crystals embedded within a glassy matrix, respond effectively to hydrofluoric acid (HF) etching due to their high silica content. Acid etching selectively dissolves the glass phase, exposing the rod-like lithium disilicate crystals, which typically measure 3–6 µm in length. The first lithia-based glass ceramic developed for use in monolithic restorations. It consists of a glassy matrix and approximately 70% crystalline content by volume, with its structure characterized by randomly oriented needle-shaped crystals ranging from 0.2 to 1 μm in size [1,2,3,4]. In contrast, zirconia, particularly 3Y-TZP is valued for its superior mechanical properties, including high strength and a transformation toughening mechanism that enhances resistance to crack propagation and fracture [5,6].

Polymer-infiltrated ceramic network (PICN) ceramics represent a class of hybrid materials with a biphasic microstructure in which HF selectively dissolves the ceramic phase, often leaving the polymer matrix more exposed. This selective etching modifies the surface morphology in ways that may enhance or hinder adhesive penetration, depending on acid strength and application time. Feldspathic ceramics and hybrid materials offer high translucency but differ in their filler content and conditioning responses. In feldspathic porcelains, HF etching produces a surface rich in micropores and microchannels, which improves bonding potential. However, excessive etching or degradation of the surface layer can lead to cohesive failure within the ceramic [7,8,9,10,11].

Various chemical, mechanical, and combination surface treatment methods have been proposed to enhance bonding effectiveness. Among them, airborne particle abrasion—typically performed through sandblasting or tribochemical silica coating using aluminum oxide (Al_2_O_3_) particles—is widely recognized [12]. This technique increases surface roughness and wettability, promotes micromechanical interlocking, and aids in surface decontamination. Increasing HF etching time or concentration raises the surface roughness in silica-based ceramics but must be balanced against the risk of strength reduction [13]. Prolonged etching—particularly beyond 90 s with HF concentrations between 10 and 20%—leads to significantly increased surface roughness but a greater probability of microcrack formation and weakening, especially in feldspathic ceramics. Untreated lithium disilicate and zirconia CAD/CAM (Computer-Aided Design/Computer-Assisted Manufacturing) blocks generally exhibit smooth surfaces, with average roughness values ranging from 0.18 to 0.22 µm after processing [14].

HF etching is effective in removing the glassy matrix of silica-based ceramics but is ineffective on zirconia due to its lack of silica content. HF concentrations between 7.5% and 10% are considered optimal, as they promote micromechanical retention by selectively dissolving the glass phase and exposing the crystalline structure without inducing excessive surface degradation [15]. Recent chemical etching protocols—particularly those using multi-acid formulations—have demonstrated the ability to achieve comparable levels of surface roughness without inducing detrimental phase transformation. These formulations typically combine hydrofluoric acid (HF) with other acids such as hydrochloric (HCl), sulfuric (H_2_SO_4_), nitric (HNO_3_), and phosphoric (H_3_PO_4_) acids, which act synergistically to degrade the oxide layer, remove surface moisture, and enhance surface roughness across various ceramic types [16].

Scanning electron microscopy (SEM) analyses show that higher HF concentrations, such as 15%, can increase etching aggressiveness but may also result in irregular porosities and compromise the material’s integrity, particularly when etching exceeds 60 s. For the HF concentrations, 10% and 15% showed mean values of SBS that were significantly higher when compared to the 1% and 2.5% values (*p* < 0.05). The concentration of 7.5% was significantly higher when compared to that of 1% (*p* < 0.05). The SEM images exhibited greater vitreous phase dissolution on the ceramic surface due to the higher HF concentration [17]. Lithium disilicate, hybrid ceramics, zirconia, and feldspathic porcelain each exhibit distinct mechanical and surface characteristics that influence their response to surface treatments such as acid etching (Table 1).

Considering these factors, it is essential to investigate how multi-acid etchants interact with different materials in different etching times. Each material—zirconia, lithium disilicate, hybrid ceramics, and feldspathic porcelain—exhibits distinct chemical and structural behaviors that influence the pattern of surface roughness during and after acid etching treatment. Systematically evaluating this novel etchant across different materials under standardized conditions will help establish optimized protocols that respect the unique properties of each material while ensuring reliable clinical performance.

Based on these considerations, the purpose of this study was to compare the surface roughness of zirconia, lithium disilicate, hybrid, and feldspathic ceramic CAD/CAM blocks following treatment with a novel multi-acid etching applied for varying durations. The null hypotheses to be tested were: (a) prolonged application of the acid etchant would not significantly increase the surface roughness of the tested materials; (b) there is no significant difference in surface roughness among the different ceramic materials following acid etching.

## 2. Materials and Methods

Chairside CAD/CAM blocks of zirconia (ZirCAD, Ivoclar Group, Schaan, Liechtenstein), lithium disilicate (Amber Mill, Hassbio, Gangneung, Republic of Korea), hybrid ceramic (CeraSmart, GC America, Alsip, IL, USA), and feldspathic porcelain (VitaBLOCS, Vita Zahnfabrik, Bad Säckingen, Germany) were sectioned using a low-speed precision cutting machine (IsoMet Low Speed Precision Cutter; Buehler, Lake Bluff, IL, USA) operating at a cutting speed of 8 mm/min. Sixty specimens were prepared for each material (number of samples = 15 per group), each with a thickness of 2 mm, yielding a total of 240 specimens.

To better simulate the intaglio surface of clinical restorations, the specimens were not subjected to finishing or polishing procedures. This approach was intentionally chosen to deviate from standard specimen preparation protocols in order to reflect more clinically relevant surface characteristics.

A detailed summary of each material’s composition, microstructure, mechanical properties, and clinical indications is provided in Table 2.

The required sample size was estimated using G*Power 3.1 based on a one-way ANOVA (fixed effects, omnibus), assuming a medium effect size (f = 0.5), an alpha level of 0.05, and a desired power of 0.90. The calculation indicated a minimum of 112 specimens distributed across 16 groups (9.3 samples per group). However, to enhance the statistical robustness, reduce standard error, and increase the reliability of the findings, the study included 15 specimens per group, totaling 240 specimens. This larger sample size also provides a margin for potential sample loss or exclusion due to technical issues, thereby strengthening the overall validity of the results.

Etching was carried out using a commercially available multi-acid etching solution formulated for zirconia (Zircos-E, EDOS, Brussels, Belgium). The solution contains a combination of hydrofluoric acid (HF), hydrochloric acid (HCl), sulfuric acid (H_2_SO_4_), nitric acid (HNO_3_), and phosphoric acid (H_3_PO_4_). These components function synergistically: HF initiates surface roughening, HCl and HNO_3_ accelerate and deepen the etching process, H_2_SO_4_ enhances the overall etching intensity, and H_3_PO_4_ facilitates debris removal, thereby optimizing the surface chemistry for bonding. To ensure consistency across specimens, etching was standardized using a micropipette to apply 25 μL of the etching solution per specimen (approximately 3 drops).

The specimens were divided into groups according to the type of ceramic material and the duration of acid application. Etching times were set at 20 s, 60 s, 30 min, and 1 h. Group details and allocations are summarized in Table 3. The selected etching times were based on manufacturer recommendations for each material: 1 h for zirconia (Zircos-E), 20 s for the lithium disilicate, and 60 s for both hybrid ceramic and the feldspathic porcelain. An additional group subjected to 30 min of etching was included to evaluate the progression of the etching reaction and to further investigate the plateau effect previously reported in the literature [16].

Following the designated etching time, all specimens were thoroughly rinsed and air-dried. To ensure the complete removal of acid residues, specimens were subsequently immersed in an ultrasonic bath (5300Weep, Quala Dental Products, Nashville, TN, USA) containing a mixture of distilled water and alcohol for 15 min. After cleaning, the specimens were stored in individual containers within desiccator for 24 h prior to surface roughness analysis. Three-dimensional (3D) surface texture and topography images were acquired using a non-contact optical profilometer (ZeGage Pro, Zygo, Middlefield, CT, USA). Additionally, two-dimensional surface morphology was analyzed using a scanning electron microscope (SEM) (Zeiss Sigma 300 VP-FESEM, Zeiss, Oberkochen, Germany).

## 3. Results

### 3.1. Surface Roughness Measurements

**Figure 1:**
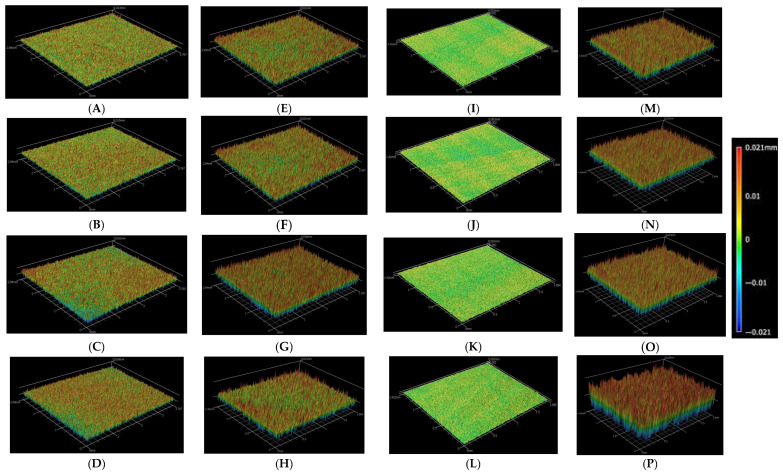
Profilometric 3D images from representative samples of each group. The green-to-red range represents positive variation (peaks), and the green-to-blue range represents negative variation (valleys). Increased roughness can be observed from top to bottom in all groups (within the same column). (**A**) Group 1 (Zirconia 20 s); (**B**) Group 2 (Zirconia 1 min); (**C**) Group 3 (Zirconia 30 min); (**D**) Group 4 (Zirconia 1 h); (**E**) Group 5 (LD 20 s); (**F**) Group 6 (LD 1 min); (**G**) Group 7 (LD 30 min); (**H**) Group 8 (LD 1 h); (**I**) Group 9 (Hybrid 20 s); (**J**) Group 10 (Hybrid 1 min); (**K**) Group 11 (Hybrid 30 min); (**L**) Group 12 (Hybrid 1 h); (**M**) Group 13 (Feldspathic 20 s); (**N**) Group 14 (Feldspathic 1 min); (**O**) Group 15 (Feldspathic 30 min); (**P**) Group 16 (Feldspathic 1 h).

**Figure 2:**
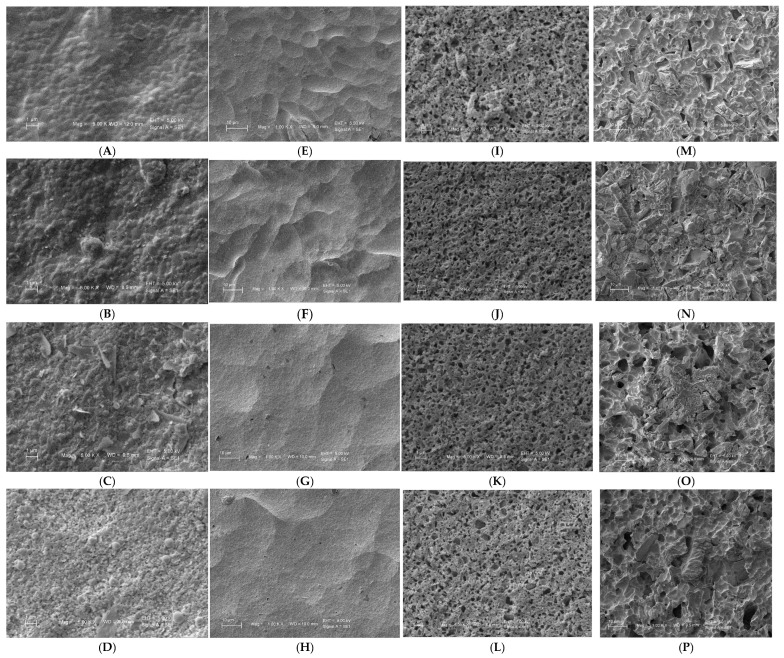
Representative scanning electron microscope (SEM) micrographs of the groups formed by material (columns) × etching time (rows). Magnification: 5000× (Z and Hy groups), 1000× (L and F groups). (**A**) Group 1 (Zirconia 20 s); (**B**) Group 2 (Zirconia 1 min); (**C**) Group 3 (Zirconia 30 min);(**D**) Group 4 (Zirconia 1 h); (**E**) Group 5 (LD 20 s); (**F**) Group 6 (LD 1 min); (**G**) Group 7 (LD 30 min); (**H**) Group 8 (LD 1 h); (**I**) Group 9 (Hybrid 20 s); (**J**) Group 10 (Hybrid 1 min); (**K**) Group 11 (Hybrid 30 min); (**L**) Group 12 (Hybrid 1 h); (**M**) Group 13 (Feld. 20 s); (**N**) Group 14 (Feld. 1 min); (**O**) Group 15 (Feld. 30 min); (**P**) Group 16 (Feld. 1 h).

Surface roughness measurements for all groups are presented in Table 4 and illustrated in Figure 1 and Figure 2. The Two-way ANOVA for the variables can be seen in Table 5. The data demonstrated that surface roughness increased proportionally with longer etching times.

The etching process altered the surface roughness differently across various materials, with etching time influencing these variations. To analyze this, a 2-way ANOVA was conducted, examining the impact of both the *ceramic material* and the *etching time*. While ranking the materials was not the primary goal, there was a statistically significant general difference in surface roughness values among the ceramic materials (*p* < 0.001). Specifically, Lithium disilicate and feldspathic specimens consistently exhibited higher average surface roughness compared to zirconia and hybrid specimens.

The etching duration significantly impacted the surface roughness of the specimens (*p* < 0.001). Longer etching times resulted in distinctly different surface characteristics when compared to shorter durations. The variables *material* and *etching time* had a noticeable interaction (*p* < 0.001). This suggests that the material type determined how etching duration affected surface roughness. Essentially, lithium disilicate and feldspathic groups confirmed this interaction effect by exhibiting a far larger increase with extended etching than zirconia and hybrid specimens. Lithium disilicate (L) and feldspathic (F) materials react more significantly to extended etching with the new solution compared to zirconia (Z) and hybrid (Hy) materials. This difference in their behavior over time explains the notable interaction observed. These comparisons highlight that, for each material, longer etching times generally lead to increased surface roughness.

**Figure 3:**
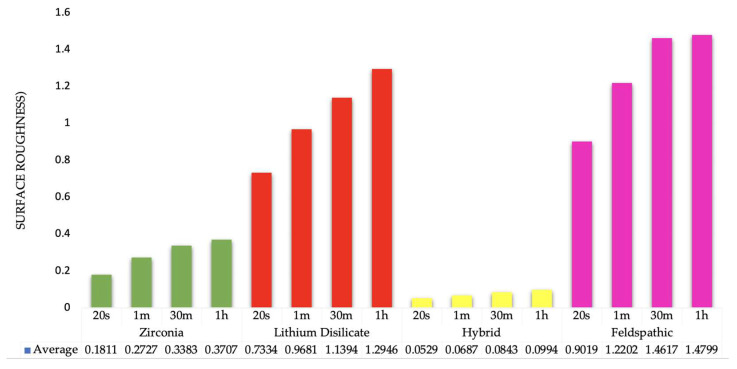
Average surface roughness (Ra) values for the zirconia (Z), lithium disilicate (L), hybrid ceramic (HY) and feldspathic (F) specimens. All specimens were treated with a zirconia etching solution for different etching times: 20 s, 60 s, 30 min, and 1 h.

For zirconia, etching for 30 min significantly increased roughness compared to 20 s and 60 s etching times (*p* < 0.001). Similarly, for lithium disilicate, a 30 min etching duration also resulted in noticeably higher roughness than both 20 s and 60 s periods (*p* < 0.001). However, this specific pattern was not observed in other materials. In general, these comparisons demonstrate that longer etching durations typically lead to increased surface roughness values across materials, with significant variations observed between control groups and different etching periods within the same material (Figure 3). Table 6 shows the pairwise post hoc comparisons illustrating the interaction between ceramic material and etching time.

### 3.2. Profilometric Imaging

Figure 1 displays profilometer observations of surfaces etched for 20 s, 60 s, 30 min, and 1 h. These three-dimensional images generally show that longer acid application led to progressively rougher surfaces. The etching process appeared to be more aggressive in the feldspathic groups (13 to 16) (Figure 1M–P). Groups with hybrid ceramic presented a less-specific etching pattern (groups 9 to 12) (Figure 1I–L).

### 3.3. Scanning Electron Microscopy (SEM)

Figure 2 presents the scanning electron microscopy (SEM) observations of samples etched for 20 s, 60 s, 30 min, and 1 h. The images clearly show increased microscopic roughness with longer durations of acid application. In general, the longer the etching time, the rougher the surface appeared, with the presence of craters, pits, and hollows. The zirconia (Figure 2A–D) and feldspathic (Figure 2M–P) groups exhibited structures resembling salt crystals. The feldspathic and the hybrid (Figure 2I–L) groups presented evidence of destruction of the structure in the 30 min and 1 h groups.

## 4. Discussion

Hydrofluoric acid (HF) etching is widely adopted for conditioning the intaglio surface of ceramic restoration, particularly glass-based ceramics, by producing a porous and irregular topography. This internal surface modification facilitates micromechanical interlocking with resin cements, thereby enhancing adhesive performance [26]. While prolonged etching times generally increases surface roughness and improves wettability, this effect eventually reaches a plateau known as “roughness saturation” where additional etching provides no further benefit. Excessive etching, or over-etching may be detrimental to bond strength due to the potential for enlarging surface flaws or compromising the ceramic’s structural integrity. Therefore, the goal is to achieve sufficient surface roughening to optimize bonding without inducing surface erosion or surpassing the saturation threshold. For multi-acid solutions such as Zircos-E, used in the treatment of 5Y-TZP, previous studies suggest that an effective etching window lies between 30 and 60 min at approximately 30 °C. Within this interval, surface roughness consistently increases before stabilizing, demonstrating the saturation behavior seen with multi-acid etching [27].

Prolonged etching, such as durations exceeding 160 s, can dissolve the surrounding glassy matrix of lithium disilicate ceramics, leading to the exposure and protrusion of the crystalline phase. This process results in elongated, randomly oriented lithium disilicate crystals, typically ranging 2.56 to 2.97 µm in length [28,29,30,31].

This behavior contrasts with that of more acid-resistant materials, such as 3Y-TZP zirconia, in which the tetragonal zirconia grains exhibit significantly lower susceptibility to acid attack. In the present study, the multi-acid etching solution appeared to induce non-selective removal of both the crystalline and glassy phases. This may be attributed to the synergistic action of the various acidic components, which likely enhanced the etching aggressiveness beyond that of hydrofluoric acid alone [32].

3Y-TZP contains approximately 3 mol% yttria, which stabilized the metastable tetragonal phase at room temperature—an essential feature contributing to its high mechanical strength and corrosion resistance. This tetragonal structure provides transformation toughening; when stressed, it converts to the monoclinic phase, and the associated volume expansion creates compressive forces that impede crack propagation. However, hydrogen ions (H^+^) leach yttrium from the lattice, destabilizing the tetragonal phase and causing its transformation into the more acid-susceptible monoclinic phase. This process causes volumetric expansion and microcracking, which exposes more surface area to the acid and accelerates dissolution [33].

In contrast, 5Y-TZP has a higher yttria content, resulting in a cubic phase that is more translucent but lacks the transformation toughening mechanism, making it more brittle. This higher yttria concentration also makes 5Y-TZP more vulnerable to yttrium leaching in acidic environments. Consequently, it is more susceptible to dissolution and fracture compared to the more robust 3Y-TZP [34].

Longer hydrofluoric acid (HF) etching times increase the surface roughness of lithium disilicate ceramics, with the average roughness (Ra) rising from 0.180 µm to 1.262 µm after 60 min of exposure. Correspondingly, Scanning Electron Microscope (SEM) images show greater surface damage with extended etching [35].

This increased roughness does not strengthen the bond because the aggressive etching solution also compromises the silica-based ceramic’s structural integrity. This damage is likely due to the material’s properties and the etchant’s high acidity.

Lithium disilicate is a glass-ceramic typically etched with hydrofluoric (HF) acid, which selectively dissolves the glassy phase to increase surface area. However, the etchant Zircos-E is significantly more aggressive. It is a highly acidic blend of several strong acids (HF, HCl, H_2_SO_4_, HNO_3_, and H_3_PO_4_) that etches more broadly than traditional HF. The combined action of its components amplifies the damage as nitric acid (HNO_3_) and hydrochloric acid (HCl) disrupt oxide bonds, while sulfuric acid (H_2_SO_4_), a strong dehydrator, alters both the glassy and crystalline phases. This synergistic action guided by HF leads to non-selective etching, which may damage the entire ceramic structure, not just the glassy matrix particularly with extended exposure, a departure from the typically selective dissolution of the glass phase expected in other silica-based ceramics. Consequently, prolonged exposure to Zircos-E can degrade or damage not only the glassy matrix but also the crystalline lithium disilicate needles. HF attacks both the glass matrix and the Li_2_Si_2_O_5_ crystalline phase, leading to the formation of SiLi_2_F_6_ precipitates on the surface of the crystals. These plate-like SiLi_2_F_6_ particles contribute to a highly irregular, nano-roughened surface, which in turn enhances both mechanical interlocking and chemical reactivity [36].

The L30 and L1h specimens exhibited significantly greater roughness than the L20 and L1m specimens (*p* < 0.001). In contrast, zirconia showed only modest increases in roughness, even with longer exposure times. This suggests that lithium disilicate is not only more effectively etched but also more susceptible to acid degradation, aligning with the “more aggressive” etching pattern observed in scanning electron microscope (SEM) images of the lithium disilicate groups.

Zirconia, which lacks a silica glass phase, is highly resistant to hydrofluoric (HF) acid etching. Short exposures (e.g., 20 or 60 s) had little impact on its surface roughness. Significant increases in roughness only occurred after 5 to 30 min, indicating zirconia’s limited susceptibility to etching and its higher chemical stability. This aligns with its polycrystalline, non-silica-based composition.

High surface roughness in lithium disilicate (LDS) is not always advantageous. Aggressive etching can create excessive roughness that compromises mechanical integrity and bonding predictability. Unlike the more chemically stable zirconia, prolonged acid exposure degrades the LDS microstructure through over-etching. This process can erode the material’s needle-like structures instead of simply exposing them, leading to suboptimal roughness and reduced micromechanical stability [37,38]. The formation of fluorinated compounds, such as SiLi_2_F_6_, provides evidence of this structural breakdown.

Zircos-E acid-etching solution effectively treats ultra-translucent zirconia surfaces by increasing roughness and altering topography without inducing a phase change. Although its initial bond enhancement may be less than sandblasting’s [39], controlled etching on 5Y-TZP preserves the material’s bulk structure and prevents excessive monoclinic conversion. After extended etching, the surface develops an irregular oxide topography as further chemical reactions plateau.

### 4.1. Surface Chemistry and Phase Composition

While higher density may seem to imply greater resistance to hydrofluoric acid (HF) etching, the ceramic’s response is more significantly governed by its crystal structure and the amount and composition of its glassy phase.

For instance, a material such as Amber Mill, with its high density and cross-linked crystal structure, will likely show a different etching pattern and degree of glassy phase removal than e.max CAD. However, the precise influence of density on etchability remains unclear and requires targeted studies under standardized conditions. The effectiveness of the etching process is also influenced by factors like HF concentration, etching time, and the ceramic’s final crystallized composition and microstructure.

### 4.2. Surface Roughness and Topography

While air abrasion typically results in higher surface roughness in zirconia compared to etching treatments, it can also produce an irregular and uneven texture. This unevenness may weaken the ceramic structure and lead to material loss. In contrast, etching creates more uniform and controlled roughness, which is sufficient to enhance bonding without compromising the integrity of the zirconia surface [40].

Cho et al. [40] specifically investigated the combination of nitric and hydrofluoric acids (HF/HNO_3_) using an etchant similar to Zircos-E, which they described as a “nitric acid–hydrofluoric acid compound” applied at room temperature for varying durations. Their finding indicated that two hours of HF/HNO_3_ etching maximized surface roughness, while one hour was less effective and three hours could be detrimental. Regarding bond strength, etching with the HF/HNO_3_ solution resulted in higher shear bond strengths to resin cements compared to air abrasion or silica coating in some resin systems. This highlights the synergistic effect of HF and HNO_3_: HNO_3_ alone would not etch, it amplifies HF’s effect to the extent that prolonged exposures can rival mechanical roughening. The clear saturation behavior observed, with a peak effect at two hours, suggests an optimal duration for HF + HNO_3_ etching.

Sokolowski et al. [41] discovered that while etching slightly lowered the average roughness parameter (Ra), this difference was not statistically significant. However, etching notably reduced the mean spacing of profile irregularities (RSm) changing it from 74.04 ± 12.74 µm for the control to 50.42 ± 13.62 µm for the etched specimens. This suggests that etching creates a denser, more uniform pattern of micro-retentions, which could improve the micromechanical bonding. The surface roughness values (Ra) were 0.6160 ± 0.0601 µm for the sandblasted (control) surface and 0.5636 ± 0.0174 µm for the sandblasted and etched surface, with no statistically significant difference between the two. Additionally, the roughness parameters were analyzed at 1000×, 5000×, and 15,000× magnification, and significant differences were observed in parameters such as the mean spacing of profile irregularities (RSm). The SEM analysis showed significant differences between the surface topography of the control and etched specimens. Etched samples had more homogeneous and evenly distributed surface irregularities. Also, chemical analysis revealed a decrease in surface aluminum concentration after etching, meaning that alumina residues were effectively removed. In conclusion, etching the zirconia surface with Zircos-E notably increased surface roughness [41].

In zirconia, increased surface roughness generally correlates positively with bond strength. This is because a rougher surface enhances the mechanical interlocking between the zirconia and the resin cement, which leads to improved adhesion and bond durability [42]. Whether this is the case when using this novel etching solution needs to be confirmed by bond strength tests.

Etched 5Y-TZP surfaces usually appear matte and frosty to the naked eye, a sign of widespread roughening. SEM micrographs can reveal a microporous network of pits and channels. These features are created by the preferential dissolution along grain interfaces and within the more acid-susceptible cubic grains. Lee et al. [43] characterized the acid-etched zirconia surface as an “excellent three-dimensional network” of micro-undercuts. Essentially, the multi-acid etch microblasts the surface: by dissolving the superficial grain structure it increases the surface area and creates micromechanical interlocks for resin infiltration.

Fluoride etching with KHF_2_ and NH_4_HF_2_ creates a rough surface morphology on zirconia, characterized by exposed zirconia grains with deep grooves and inter-granular porosity, which facilitates mechanical interlocking and enhances bonding strength. These etching processes result in a surface similar to HF-etched porcelain, improving adhesion of resin-based cements. Bond strength values after melt etching with these fluorides are comparable to or better than sandblasting, especially when combined with silanization, indicating improved chemical and mechanical bonding [44].

SEM images show that Zircos-E etching creates progressively greater etching pattern. Porosity is significant after 30 min, and a dense microporous structure covers the surface by 60 min. Because further etching primarily deepens existing pits rather than forming new ones, the process plateaus. Essentially, surface roughness increases quickly before “saturating” as the etching time nears one hour under typical conditions.

A brief 30 s etch is largely ineffective on inert zirconia, providing little more than a surface cleaning. significant roughness and porosity (Ra ≈ 1 µm or more) only begin to appear after several tens of minutes of acid exposure. The acid blend brings the surface to a quasi-steady state of roughness and chemical activation in about an hour. Therefore, clinicians gain little by etching longer than 60 min but must etch for at least a few tens of minutes to achieve a clinically meaningful result.

The synergistic multi-acid etching of 5Y-TZP presents a double-edged sword as it is highly effective at creating a bondable surface, especially within an optimal timeframe, but it demands careful control to prevent plateaus or negative effects from over-etching or residues [16]. When performed correctly, Zircos-E etching improves micromechanical retention and maintains surface integrity. This makes it a viable chemical alternative or addition to traditional airborne abrasion for achieving durable resin-zirconia bonds.

### 4.3. SEM Analysis

Acid etching produces a uniform, microporous surface with tiny grooves, which improves the surface for bonding without resorting to aggressive roughening. In this study, SEM analysis revealed that 60 min of etching changed the morphological characteristics, particularly in highly translucent specimens, leading to a more pronounced surface irregularity. These microstructural changes are notable with scanning electron microscopy, confirming these characteristic surface features [45].

The findings of this study are partially supported by other SEM examinations, which show distinct variations across the groups. Only the Z1h group displayed markedly roughened and porous surfaces with clear micro-retentions, formed by the selective dissolution of zirconia grains.

### 4.4. Influence of Ceramic Composition

The hybrid material tested (CeraSmart, GC) is a hybrid ceramic, available as CAD/CAM blocks, known for its strength and flexibility. It is made of a flexible nano-ceramic matrix with dispersed nano-ceramic particles and a composite resin that contains 71% silica and barium glass nanoparticles by weight.

The recommended concentration of hydrofluoric acid (HF) for CeraSmart is 2.0% for 1 min. Alternatively, a 30 s etch with 9% HF has proved to be effective without causing surface damage. Since the exact concentration of the components of Zircos-E is still not disclosed, one can assume that the concentration of HF is Zircos-E Etchant is not strong enough to affect significantly the silicon content of CeraSmart. HF etching at higher concentrations (around 9.5%) significantly decreases the silicon content due to reaction with Si [46]. Whether the increase in surface roughness is suitable for adhesion or not, it will be the subject of future investigations. Another possible explanation for the moderate effect of the etchant is the composition of CeraSmart. The barium glass (300 nm) nanoparticles are more resistant to etching with HF than other species of glasses. Its reaction with HF produces a precipitate of BaF_2_, that remains as a crystalline residue on the surface or can be rinsed off, depending on etching protocol and solubility conditions. Its densely polymerized matrix (29 wt.% Bis-MEPP polymer) is more resistant to the action of the HF [47].

The long-term effects of using self-etching primers versus hydrofluoric acid (HF) etching on ceramic-resin bond durability are still under investigation, but current evidence suggests that self-etching primers can provide comparable bond stability with less surface damage and lower toxicity. Studies indicate that self-etching primers may maintain bond strength effectively over time, especially when combined with appropriate silanization, and may exhibit better clinical performance due to reduced surface degradation and fewer microstructural alterations caused by HF etching. However, HF etching, while initially producing higher surface roughness and bond strength, may lead to surface degradation over time, potentially compromising long-term durability [48]. About using airborne abrasion, a recent study found that the application of HF after airborne-particle abrasion resulted in similar contact angle values as when only HF was applied [49].

A limitation of this study is that it focused exclusively on surface roughness without evaluating the mechanical performance of the etched ceramic, such as bond strength. Future investigations should include mechanical testing to assess the adhesive performance of various resin cements in combination with different ceramics treated using this multi-acid etching protocol. Nonetheless, the surface roughness data presented here provide a fundamental understanding that can guide the selection of optimal etching time for the subsequent bond strength studies.

Further research should also explore the integration of chemical bonding strategies with mechanical roughening, alongside the use of resin cements, as this approach generally produces the most reliable and durable bonds. Continued advancements in this area are essential to improve clinical outcomes by optimizing both bond strength while maintaining the structural integrity and long-term stability of ceramic restorations.

## 5. Conclusions

The alternative etching method effectively increased the surface roughness of the zirconia and lithium disilicate specimens. While acknowledging the role of chemical adhesion in bonding porcelain to zirconia, it can be concluded that etching primarily enhances mechanical retention. This highlights the critical balance needed in surface treatments, as over-etching could negatively impact zirconia strength and structural integrity. The plateau in roughness values and minimal damage observed under SEM suggest that within the times tested (20 s, 1 min, 30 min, and 1 h) the 1 h treatment may be considered as optimal, providing enhanced surface roughness for zirconia surfaces without the adverse effects observed at longer etching durations. The novel zirconia etching solution provided non-selective etching of lithium disilicate surfaces, probably due to the synergistic action of the components.

## Figures and Tables

**Table 1:** Acid etching time with hydrofluoric acid (HF) for dental ceramics [18,19,20,21,22].

Type of Ceramic	HF Application Time
Zirconia (ZirCAD, Ivoclar)	Not recommended
Lithium Disilicate (Amber Mill, Hassbio)	20 s
Hybrid Ceramic (CeraSmart, GC)	60 s
Feldspathic Porcelain (VitaBLOCS, Vita)	60–90 s

**Table 2:** Ceramic materials used, categorized by their microstructure, physical characteristics, and applications. σ (flexural strength), Ra (surface roughness), KIc (fracture toughness), E (elastic modulus), and CTE (coefficient of thermal expansion) [22,23,24,25].

Material	Commercial Brand	Crystalline Microstructure	Mechanical Properties CTE	Clinical Indication
Zirconia [1]	e.max ZirCAD^®^ (Ivoclar) Block size: 12 × 14 × 18 mm	Homogeneous fine Y_2_O_3_: 4.0–6.0 ZrO_2_: 87.0–95.0% HfO_2_: 1.0–5.0, Al_2_O_3_: 0.0–1.0 Other oxides: <0.2	σ: ≥900 MPa Ra: 0.22 µm KIc: 5.14 ± 0.07 MPa·m^1/2^ E: 70 ± 2 GPa CTE: 10.6 ± 0.1 × 10^−6^ K^−1^ (100–400 °C)	Full-contour crowns, 3-unit bridges, and 4- and multi-unit bridges with max. 2 pontics, Crown copings, 3 unit- and multi-unit bridge frameworks with max. 2 pontics
Glass-Ceramic Lithium disilicate [1]	Amber Mill^®^ (Hassbio) Block size: 12 × 14 × 18 mm	Needle–like crystals (approx. 70 vol%); Composition: Li_2_Si_2_O_5_; Size: 3–6 µm (length)	σ: 350–450 MPa Ra: 0.21 µm KIc: 0.8–1.5 MPa·m^1/2^ E: ~70 GPa CTE: 10.2 ± 0.4 × 10^−6^ K^−1^ (100–400 °C), 10.6 ± 0.35 × 10^−6^ K^−1^ (100–500 °C)	Crowns, Veneers Occlusal veneers (table tops) ≥ 1.0 mm, Inlays, Onlays, Partial crowns, 3-unit bridges in the anterior and posterior region (2nd premolar as the terminal abutment) Hybrid abutments in the anterior and posterior region as a single-tooth restoration, Hybrid abutment crowns in the anterior and posterior region
Hybrid Ceramic	CeraSmart (GC America) Block size: 12 × 14.0 × 18 mm	~71–80% ceramic nanoparticles (silica + zirconia) in a resin matrix (UDMA, Bis-MEPP, Bis-GMA) Highly dispersed nano-sized fillers bonded in a resilient resinous matrix	σ: ~220 MPa KIc: ~1.2–1.6 MPa·m^0.5^ E: ~10–15 GPa Sa ≈ 0.05–0.15 µm (post-polishing) CTE: ~30–50 × 10^−6^/°C (resin-like behavior) ~1.0 GPa (Vickers)	- Inlays, onlays, veneers - Single-unit posterior crowns - Implant-supported restorations (short-term) - Ideal for bruxers and high-load patients - Suitable for same-day dentistry (no firing) - Resin-based structure allows easy repair
Feldspathic Ceramic	VitaBLOCS, (Vita) Block size: 10 × 12 × 15 mm	Feldspathic ceramic Glass matrix with fine feldspar crystals (~60–70 vol% crystalline phase) Homogeneous crystalline-glass microstructure CAD/CAM blocks, sintered glass-ceramic	σ: ~130–160 MPa KIc: ~1.1–1.5 MPa·m^0.5^ E: ~45–65 GPa Sa ≈ 0.15–0.3 µm (depends on finish) CTE: ~9–10 × 10^−6^/°C ~5.5 GPa (Vickers)	- Inlays, onlays, veneers - Anterior and premolar crowns (posterior use limited) - Esthetic veneers and crowns in low-stress areas - Ideal for anterior esthetics and layering with enamel - Requires polishing, firing not always mandatory - More brittle; repairs less predictable

**Table 3:** Group distribution according to the material tested and etching time (n = 15).

	Zirconia (Z)	Lithium Disilicate (L)	Hybrid (Hy)	Feldspathic (F)
Etching time				
20 s	Group 1 (Z20)	Group 5 (L20)	Group 9 (Hy20)	Group 13 (F20)
1 min	Group 2 (Z1m)	Group 6 (L1m)	Group 10 (Hy1m)	Group 14 (F1m)
30 min	Group 3 (Z30)	Group 7 (L30)	Group 11 (Hy30)	Group 15 (F30)
1 h	Group 4 (Z1h)	Group 8 (L1h)	Group 12 (Hy1h)	Group 16 (F1h)

**Table 4:** Mean values of surface roughness according to the material and the application time for the different groups.

Application Time	Mean Surface Roughness in Microns (µm) (Standard Deviation) ^1^			
	Zirconia	Lithium Disilicate	Hybrid	Feldspathic
20 s	Group 1 0.1811 (0.043)	Group 5 0.7334 (0.082)	Group 9 0.0528 (0.008)	Group 13 0.9019 (0.102)
1 min	Group 2 0.2727 (0.041)	Group 6 0.9680 (0.066)	Group 10 0.0686 (0.007)	Group 14 1.2202 (0.166)
30 min	Group 3 0.3382 (0.037)	Group 7 1.1394 (0.098)	Group 11 0.0843 (0.006)	Group 15 1.4617 (0.100)
1 h	Group 4 0.3706 (0.074)	Group 8 1.2946 (0.123)	Group 12 0.0994 (0.016)	Group 16 1.4799 (0.096)

^1^ standard deviation between parentheses.

**Table 5:** Two-way ANOVA for the variables material (zirconia, lithium disilicate, hybrid material, and feldspathic porcelain) and time (20 s, 1 min, 30 min, and 1 h).

	Sum of Squares	df	Mean Square	F	Sig.
Between Groups	65.250	15	4.350	664.323	<0.001
Within Groups	1.467	224	0.007		
Total	66.717	239			

Note: type III sum of squares; df: degrees of freedom, F: F-distribution, Sig.: Level of significance.

**Table 6:** Pairwise post hoc comparisons illustrating the interaction between ceramic material and etching time. Within each column and row, different letters denote statistically significant differences (*p* < 0.05).

Etching Time	Group Number (Standard Deviation)			
20 s	(Group 1) 0.181 (0.043) b	(Group 5) 0.733 (0.082) d	(Group 9) 0.053 (0.008) a	(Group 13) 0.902 (0.102) d
1 min	(Group 2) 0.273 (0.041) b	(Group 6) 0.968 (0.066) d	(Group 10) 0.069 (0.007) a	(Group 14) 1.220 (0.166) d
30 min	(Group 3) 0.338 (0.037) b	(Group 7) 1.139 (0.098) d	(Group 11) 0.084 (0.006) a	(Group 15) 1.461 (0.100) d
1 h	(Group 4) 0.371 (0.074) c	(Group 8) 1.295 (0.123) d	(Group 12) 0.099 (0.016) a	(Group 16) 1.479 (0.096) d

## Data Availability

Data presented in this study are available on request from the corresponding authors.
